# Clinicians’ prescription preferences for treating patients with Alzheimer’s disease in Shanghai

**DOI:** 10.1186/s40035-016-0055-3

**Published:** 2016-04-25

**Authors:** Chun-Xia Ban, Shi-Fu Xiao, Xiang Lin, Tao Wang, Qi Qiu, Min-Jie Zhu, Xia Li

**Affiliations:** Alzheimer’s Disease and Related Disorders Center, Department of Geriatric Psychiatry, Shanghai Mental Health Center, Shanghai Jiao Tong University School of Medicine, Shanghai, China; Mental Health Center of Jiading District in Shanghai, Shanghai, China

**Keywords:** Alzheimer’s disease, Clinicians, Cognitive enhancers, Prescriptions, Survey research

## Abstract

**Background:**

China has more cases of Alzheimer’s disease (AD) than any other country in the world. As training to recognize and manage dementia is in its early stage, it is important to study clinicians’ current prescription preferences for treating patients with AD.

**Methods:**

This study surveyed neurologists, psychiatrists, and general physicians (GPs) in Shanghai who had outpatients with AD, using a questionnaire asking about their prescription preferences for these patients.

**Results:**

Among the 148 clinicians in the study, 26.4 % were psychiatrists, 44.6 % were neurologists, and 29.1 % were GPs. The groups did not differ significantly in age, gender, or their monthly cases of new patients with mild or moderate AD (*P* > 0.05). Most clinicians prescribed Cholinesterase inhibitors (ChEIs), including Huperzine A, but there were significant group-differences in prescribing specific ChEIs (*P* < 0.05). The daily dosages of ChEI and Memantine prescribed by all three groups were small (*P* > 0.05), and all three groups prescribed piracetam, ergot, and ginkgo biloba drugs. All three groups also tended to treat AD patients with a combination of antidepressants and anxiolytics, although psychiatrists were significantly more likely than neurologists to combine antipsychotics with other drugs (*P* < 0.05).

**Conclusion:**

Clinicians in Shanghai prescribed low doses of ChEIs and Memantine for patients with AD. A relatively high proportion also prescribed cognitive enhancers, which lack evidence-based support of their use, and antipsychotics. There is a need for more training about treating patients with AD and for clinicians to standardize their clinical practice.

## Background

Alzheimer’s disease (AD) is a chronic neurodegenerative disease that has become one of the largest public health problems. Over 25 % of all people with AD are living in China [1]. In 2010, China had 5.69 million cases of AD, which was more than any other country in the world [2]. With the accelerated growth of the older population, the number of people with AD is estimated to triple by 2050 [3]. Although there is no current cure for AD, appropriate treatment can benefit the elderly with AD and improve their quality of life. Medication is one of the most important interventions [4].

Worldwide, only three cholinesterase inhibitors (ChEIs)—Donepezil, Rivastigmine, and Galantamine—and the NMDA-receptor antagonist Memantine are recommended for managing AD. Compared to other developing countries, these medicines were approved later by the Chinese government, so they became available later for Chinese patients. Nevertheless, another ChEI, Huperzine A, which was developed by Chinese scientists, has been used for treating AD in clinical practice for over thirty years [5]. In the USA, Huperzine A is a nutritional supplement for enhancing memory.

In China, training for the recognition and management of dementia is in the early stage [6]. The Chinese guidelines for the treatment of AD are not necessarily taught to clinicians. Furthermore, memory clinics have been developed only in recent years. Given this background, it is worthwhile to examine how clinicians in China prescribe medications for patients with AD.

In many countries, such as the USA, Australia, Canada or European countries, patients with AD are usually treated first by family doctors—general physicians (GPs); 39 % of all patients with AD are treated by the departments of neurology, psychiatry, and other departments [7]. Because the Chinese medical system has its own characteristics, such as the lack of referrals between general and specialized departments, the clinical treatment of patients with AD is mainly done by the department of neurology, the department of psychiatry, and the general department (including internists at district hospitals and community physicians) without referrals. There has been no investigation, to date, on how clinicians prescribe medications for patients with AD, to see if there are similarities and differences in the medications prescribed by neurologists, GPs, and psychiatrists, and whether the medication principles of these three groups are in accordance with popular diagnostic and management manuals, such as EFNS guidelines for the diagnosis and management of Alzheimer’s Disease (EFNS, 2010) [8]; Current pharmacologic treatment of dementia: a clinical practice guideline from the American College of Physicians and the American Academy of Family Physicians (2008) [9]; Practice Guideline for the Treatment of Patients With Alzheimer’s Disease and Other Dementias (APA,2007) [10].

Shanghai, which is the largest city in China, has had rapid cultural and economic development. Moreover, the increase in Shanghai’s aging population has been ahead of that in other cities. We developed a questionnaire to ask clinicians in Shanghai, who have outpatients with AD or cognitive disabilities, about their prescription preferences and the characteristics of their patients with AD in order to understand the current status of the diagnosis and treatment of patients with AD. The results can provide useful insights for developing relevant training and public health policies for the future.

## Methods

### Subjects

All the psychiatrists, neurologists, and GPs from community hospitals, second-tier hospitals, and tertiary hospitals who had patients with dementia in Shanghai were enrolled in the study from September 2012 to March 2013. The inclusion criteria were: (1) physicians who specialized in the diagnosis and treatment of AD; and (2) held a professional clinical position of attending doctor or above. The exclusion criteria were: (1) clinicians who did not have independent medicine prescriptive authority; (2) clinicians who treated less than five AD patients each month; (3) clinicians who did not complete half of the items on the questionnaire; and (4) clinicians who did not sign the consent form. This research was approved by the Ethical Review Board of the Shanghai Mental Health Center of Shanghai Jiaotong University.

The nature and aim of the study were explained to the potential participants. Clinicians who agreed to participate signed the informed consent form, and then they were instructed to complete an 18-item self-administered questionnaire. In total, 172 questionnaires were collected. We excluded 7 clinicians who never diagnosed or treated patients with AD, and 17 clinicians who did not complete the survey. leaving 148 physicians who were included in the analyses. There were 74 males (50.0 %) and 74 females (50.0 %), ranging in age from 26 to 60 years. Their mean age was 42.01 ± 7.936 years. The percentages of psychiatrists, neurologists, and GPs were 26.4 % (39/148), 44.6 % (66/148), and 29.1 % (43/148), respectively.

### Measures

We developed a questionnaire that included three sections of questions. The three sections were: (1) prescription preferences for ChEIs and Memantine; (2) other prescription preferences for cognitive enhancers; and (3) prescription preferences for antidepressants and anxiolytics. The other types of information collected by the questionnaire were: age, gender, the clinician’s department and hospital; the number of new cases each month involving the diagnosis and treatment of patients with AD; the proportion of treated patients with mild, moderate, or severe AD; the proportion of patients with AD using antidepressants, antipsychotics, anxiolytics, or sedative-hypnotic drugs; the proportion of agreement on using one type of ChEIs; preferences and reasons for patients with AD using a certain type of ChEI; reasons why patients with AD did not use a certain type of ChEI; the proportion and daily doses of patients with AD using Donepezil, Rivastigmine, Galantamine, and Huperzine A; the proportion and daily doses of patients with AD using Memantine, and the reasons for using it; the proportion of, and the daily doses, of patients with AD using combination of ChEIs and Memantine, and the reasons for using them in combination; the proportion of patients with AD using Qxiracetam/Aniracetam, ginkgo biloba extract (EGB), ergot alkaloids, vitamin E, nutritional supplements, herbs/traditional Chinese medicine (TCM); and, whether clinicians prescribed the medications described above for patients with AD. The questionnaire generally took 10 to 20 min to complete.

### Statistical analysis

We checked and proofread the collected data and analyzed the data using SPSS software. Continuous data with normal distributions, including age, the number of new cases diagnosed per month, and the percentage of patients with different mild, moderate, and severe AD were expressed as the mean ± the standard deviation, and analyzed by one-way analysis of variance. Categorical data were expressed as percentages, including gender, the proportion of prescriptions for ChEIs, Donepezil, Rivastigmine, Galanthamine, Huperzine A, and Memantine, the reasons for choosing ChEIs and Memantine, and the prescription rates of other drugs. The comparison of the proportion of prescriptions of ChEIs was analyzed by Fisher’s exact test and other comparisons of proportions were analyzed by the chi-square test. The proportion of prescriptions of antipsychotics, antidepressants, and anxiolytics among the three groups of clinicians were expressed as the median (interquartile range); their distributions were assessed for normality by the Shapiro-Wilk test and their homogeneity of variance was assessed by Levene’s test. If the data were not normally distributed, the non-parametric Kruskal-Wallis test for comparing medians was used, and the Mann–Whitney U-test was used for multiple comparisons among the different groups. The significant level was set at α = 0.05, using two-tailed tests. Results that showed *P* < 0.05 were considered to be statistically significant.

## Results

### Characteristics of study participants

The characteristics of the clinicians who completed the survey are shown in Table 1. There was no statistical difference among the psychiatrists, neurologists, and GPs with respect to age, gender, the numbers of new cases diagnosed per month, or the proportion of cases with different levels of AD severity (the percentage of patients with mild, moderate, and severe AD) (*P* > 0.05).

**Table 1:** Characteristics of the Study Participants

Characteristic	Psychiatrists (*n* = 39)	Neurologists (*n* = 66)	General Physicians (*n* = 43)	*F/X* ^*2*^	*P* value
Age,mean(SD)	41.87(8.36)	41.17 (7.38)	43.44 (8.34)	1.080	0.342
Gender,n (%)					
Male	22 (56.40)	32 (48.50)	20 (46.50)	0.911	0.634
Female	17 (43.60)	34 (51.50)	23 (53.50)		
Numbers of new cases which are diagnosed per month, mean(SD)	14.74(14.90)	13.26(16.04)	13.67(17.20)	0.105	0.900
Severity of AD (%)					
Mild, mean(SD)	31.95(20.60)	34.60(22.64)	37.09(23.48)	0.541	0.584
Moderate, mean(SD)	39.62(16.50)	40.17(20.46)	36.05(13.95)	0.753	0.473
Severe, mean(SD)	28.44(18.35)	25.24(18.09)	26.63(17.24)	0.391	0.677

### Rates of prescribing ChEIs and Memantine

Most psychiatrists, neurologists, and GPs prescribed ChEIs to treat patients with AD (see Table 2). The three most commonly used ChEIs were, respectively: Donepezil (90.9 %), Huperzine A (68.2 %), and Rivastigmine (27.3 %) by neurologists; Huperzine A (87.2 %), Donepezil (51.3 %), and Galantamine (17.9 %) by psychiatrists; and Huperzine A (65.1 %), Donepezil (48.8 %), Rivastigmine (9.3 %) by GPs. There were significant differences in the percentages of psychiatrists, neurologists, and GPs choosing ChEIs agents (*P* < 0.05, see Table 2).

**Table 2:** Rates of Prescribing ChEIs and Memantine

Drug Characteristics	Psychiatrists (*n* = 39)	Neurologists (*n* = 66)	General physicians (*n* = 43)	*F/X* ^*2*^	*P* value
ChEIs, n (%)	37(94.9)	65(98.5)	41(95.2)	1.605	0.601
Donepezil, n (%)	20(51.3)	60(90.9)	21(48.8)	28.295	0.000
Dose (mg/day), mean(SD)	5.75 ± 1.832	5.67 ± 1.714	5.24 ± 1.091	0.651	0.524
Rivastigmine, n (%)	1(2.6)	18(27.3)	4(9.3)	13.200	0.001
Dose (mg/day), mean(SD)	6.00 ± 0.000	5.83 ± 2.515	5.25 ± 2.872	0.090	0.915
Galanthamine, n (%)	7(17.9)	11(16.7)	1(2.3)	6.022	0.049
Dose (mg/day), mean(SD)	15.43 ± 2.760	14.91 ± 4.764	12.00 ± 0.000	0.303	0.743
Huperzine A, n (%)	34(87.2)	45(68.2)	28(65.1)	5.978	0.050
Dose (ug/day), mean(SD)	280.88 ± 81.66	263.33 ± 89.443	278.57 ± 95.674	0.456	0.635
Memantine, n(%)	8(20.5)	39(59.1)	9(20.9)	22.878	0.000
Dose (mg/day), mean(SD)	11.88 ± 4.581	11.15 ± 4.209	11.67 ± 2.500	0.140	0.870

Memantine was used by 20.5 % of psychiatrists, 59.1 % of neurologists, and 20.9 % of GPs for treating AD. The rate of neurologists who prescribed Memantine was higher than the rate among psychiatrists and GPs (*P* < 0.01, see Table 2). There was no statistically significant difference among psychiatrists, neurologists, and GPs in their daily prescribed dosages of ChEIs and Memantine (*P* > 0.05, see Table 2).

### Reasons for choosing ChEIs and Memantine

Regarding the reasons why clinicians prescribed ChEIs: 71.9 % of physicians agreed that ChEIs were effective, 35.9 % considered them safe, 10.9 % thought they were familiar with ChEIs, and 9.4 % of them used ChEIs based on support for ChEIs from evidence-based research. Other reasons for choosing ChEIs included convenience for patients to take them orally (once per day), the guidelines’ recommendations, ChEIs being the only available AD medication in the hospital, and their ability to control behavioral and psychological symptoms of dementia (BPSD). In all, 37.8 % of physicians prescribed Memantine for treating patients with AD; 47.8 % prescribed Memantine for patients with moderate or severe AD; 19.6 % chose Memantine to control BPSD, and 13.0 % used Memantine when ChEIs had an inadequate effect on patients. Other reasons why physicians prescribed Memantine included fewer side-effects, patients having contraindications to ChEIs, combined use with ChEIs, and support from evidence-based research (see Table 3).

**Table 3:** Reasons for Choosing ChEIs and Memantine

Reasons for choosing ChEIs	Proportions of clinicians (*n* = 64)	Reasons for choosing memantine	Proportions of clinicians (*n* = 64)
Effectiveness	46 (71.9 %)	moderate or severe AD	22 (47.8 %)
Safety	23 (35.9 %)	controlling BPSD	9 (19.6 %)
Familiar with ChEIs	7 (10.9 %)	poor response to ChEIs	6 (13.0 %)
Support of evidence-based research	6 (9.4 %)	effectiveness	6 (13.0 %)
Convenience for patients oral taking	5 (7.8 %)	fewer side-effect	3 (6.5 %)
Guidelines’ recommendation	4 (6.3 %)	Patients had contraindications to ChEIs	2 (4.3 %)
The only available AD medication in the hospital	3 (4.7 %)	combination use with ChEIs	1 (2.2 %)
Controlling BPSD	1 (1.6 %)	support of evidence-based research	1 (2.2 %)

### Rates of prescribing other drugs

When diagnosing and treating patients with AD, 56.4 % of psychiatrists, 65.2 % of neurologists, and 69.8 % of GPs prescribed Oxiracetam/Aniracetam; 71.8 % of psychiatrists, 72.7 % of neurologists, and 79.9 % of GPs prescribed ginkgo biloba extract; 46.2 % of psychiatrists, 57.6 % of neurologists, and 41.9 % of GPs prescribed ergot alkaloid; 10.3 % of psychiatrists, 37.9 % of neurologists, and 53.5 % of GPs prescribed vitamin E; 17.9 % of psychiatrists, 60.6 % of neurologists, and 58.1 % of GPs prescribed nutritional supplements; and 28.2 % of psychiatrists, 50.0 % of neurologists, and 53.5 % of GPs prescribed herbs/traditional Chinese medicine.

There were significant differences in the percentages of the psychiatrists, neurologists, and GPs prescribing vitamin E, nutritional supplements, and herbs/traditional Chinese medicine (*P* < 0.05, see Table 4).

**Table 4:** Percent of Clinicians Prescribing Other Drugs

	Psychiatrists (*n* = 39)	Neurologists (*n* = 66)	General physicians (*n* = 43)
Oxiracetam/aniracetam n (%)	22(56.4)	43(65.2)	30(69.8)
Ginkgo Biloba extract n (%)	28(71.8)	48(72.7)	34(79.1)
Ergot alkaloid n (%)	18(46.2)	38(57.6)	18(41.9)
Vitamin E n (%)**	4(10.3)	5(37.9)	223(53.5)
Nutrition supplements n (%)**	7(17.9)	40(60.6)	25(58.1)
Herbs/traditional Chinese medicine n (%)*	11(28.2)	33(50.0)	3(53.5)

Notes: *P < 0.05, **P < 0.01

### Rates of prescribing antipsychotics, antidepressants, and anxiolytics

**Fig. 1:**
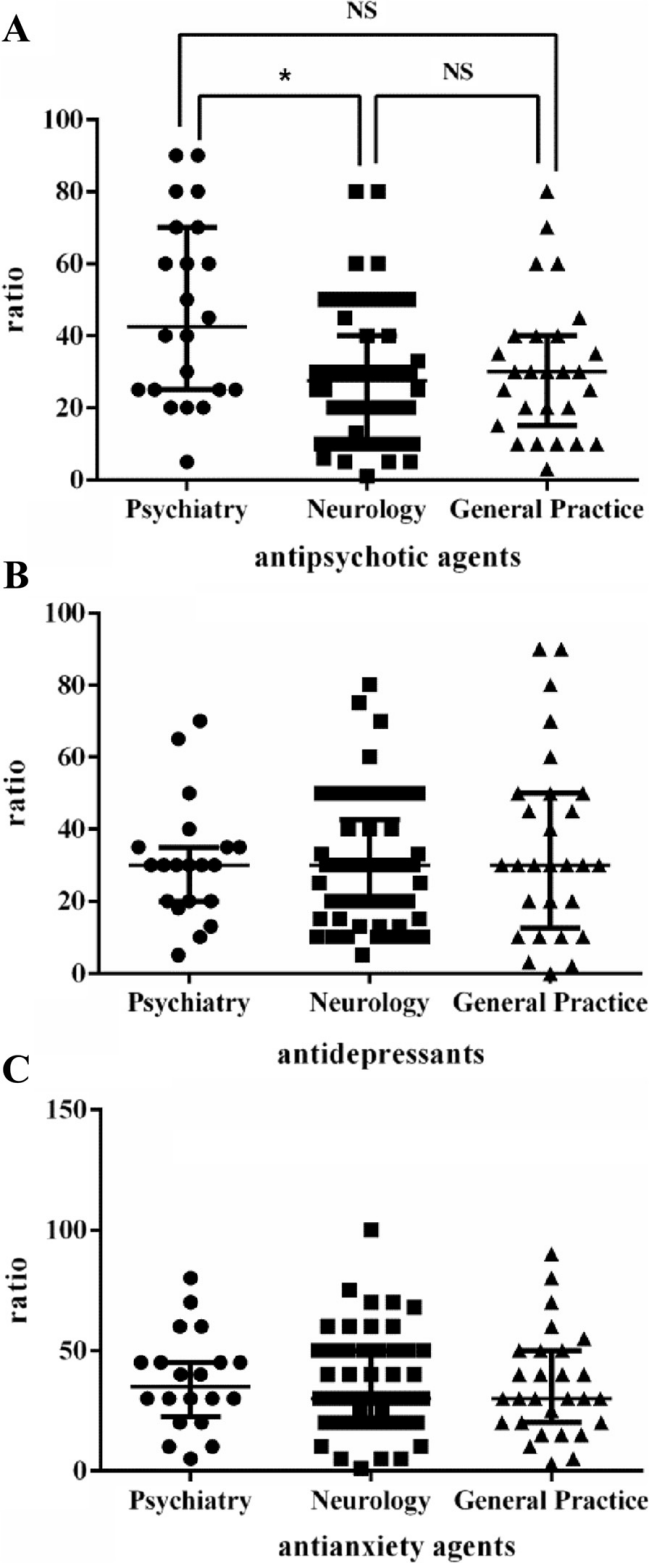
Percentages of the three group of clinicians who prescribed antipsychotics, antidepressants, and anxiolytics

There was a significant difference among the groups in terms of prescribing antipsychotics. A higher proportion of psychiatrists prescribed antipsychotics for patients with AD than neurologists or GPs. This difference was statistically significant between psychiatrists and neurologists (*P* < 0.05, see Fig. 1).

There was no significant difference among the three groups of clinicians with respect to the proportion who prescribed antidepressants or anxiolytics for patients with AD (*P* > 0.05, see Fig. 1).

## Discussion

Alzheimer’s disease (AD) is a disease with high rates of disability that have a great burden. There are nearly 44 million patients with AD worldwide, and it is estimated that the number will increase to 135 million by 2050 [11]. The number of patients with AD in China was 3.71 million in 2000, and 5.69 million in 2010 [2], and it is estimated that the number of patients with AD in China will increase to 27 million in 2050 [12]. All these numbers show rising trends. Because of the one-child policy and the internal migration policy in China, patients with dementia lack caregivers and will have serious economic burdens [12].

The EFNS and APA guidelines all suggest that clinicians use ChEIs (Donepezil, Rivastigmine, and Galantamine) and Memantine as first-line medications for treating patients with AD [10, 8]. Clinicians, today, still mainly use ChEIs to treat symptoms of patients with AD [13], and those medications have been shown to be clinically effective and safe [14]. Clinical practice guidelines published by the APA, the American College of Physicians (ACP), and the American Academy of Family Physicians (AAFP) all have noted the effectiveness and safety of using ChEIs [10, 9]. Memantine is a NMDA receptor antagonist that is approved for treating patients with moderate or severe AD [10]. Our results showed that over 94 % of clinicians in each group considered using ChEIs because of its effectiveness and safety. There were low proportions of clinicians in the three groups who chose Memantine, some of whom chose it because patients had moderate or severe AD. Therefore, the study’s results are in line with the prescription recommendations given by clinical guidelines.

We should mention that there are special characteristics and circumstances in China. First, Chinese patients who follow clinicians’ prescriptions mostly obtain their medications at the dispensary of the clinician’s hospital, and every hospital’s dispensary provides different kinds of medications. Dispensaries in tertiary hospitals have a relatively comprehensive range of medications, and they accept relatively new kinds of drugs. The dispensaries of community hospitals have the fewest kinds of drugs and provide relatively basic and inexpensive medications. The situation of second-tier hospitals is between that of the community hospitals and the tertiary hospitals. Second, Donepezil was approved by the FDA for use in public clinical practice in 1996, and it has been used in clinical practice in Shanghai since 2000. However, it was not until recently that Donepezil has been included in medicare reimbursement in Shanghai and other regions. Furthermore, Donepezil and Memantine are only available at some hospitals’ dispensaries. The last characteristics is that Huperzine A, which is extracted from Huperzia serrata (a traditional Chinese medicine), is a new type of sesquiterpene alkaloid compound [5].

Huperzine A was independently developed by Chinese scientists, and it appeared on the Chinese market for treating patients with AD in 1995. Huperzine A was marketed much earlier than Donepezil and Memantine, and the price of Huperzine A is cheaper than these two drugs. If we calculate the daily dose of Huperzine A as 300 μg, the price of it is approximately 15 % of a 5 mg dose of Donepezil. Huperzine A is available in nearly every hospital in China. Because of the characteristics described above, the present study included neurologists, who were mostly from tertiary hospitals, psychiatrists, who were mainly from second-tier hospitals—a few were from tertiary hospitals (such as Shanghai Mental Health Center), and GPs, who were mainly from community hospitals (see Appendix). Thus, the results showed that most neurologists chose Donepezil and Memantine, and most psychiatrists and GPs chose Huperzine A. These findings may be related to the availability of different medications in different kinds of hospitals. The low prescription rates of Memantine may be that it was marketed in China late in 2006 and lack of availability at hospitals. In the near future, when patients in China could choose their medicine allocation, a further investigation on clinicians’ prescription would be updated.

A previous study showed that the ChEIs dosage is related to its effects [15], and another study indicated that the administration of Memantine should be given in adequate doses [16]. The present study found that the medication doses of Memantine and ChEIs were both low. One reason for this may be that clinicians and patients did not have enough knowledge about AD. Li and her colleagues [17] investigated community residents in Shanghai and found that people had little understanding about the early phase of AD and the benefits of treatment. Under these circumstances, clinicians might not be very positive about treatment, thus, leading to low doses of medications. A second reason may be that clinicians did not have ready access to new theories and medications in their specialties. Some clinicians were not familiar with cognitive enhancers and had little knowledge about related theories and medications. The last reason might be the price of the drugs. Patients with AD have a huge economic burden, and medicine costs are one part of this burden. Most ChEIs and Memantine, which are approved by the FDA, are all expensive at present. Hence, patients who use their own money to buy drugs may choose lower doses or even stop treatment because of the price. Besides, medicare reimbursement in China has regulations that prevent clinicians from prescribing high priced medications. Therefore, even for patients who have medicare reimbursement, their clinicians may prescribe relatively low doses of ChEIs and Memantine.

The study showed that relatively high proportions of the three groups of clinicians used Oxiracetam/Aniracetam, ginkgo biloba extract, and ergot alkaloid. These medications do not have sufficient support from evidence-based research, which leaves their treatment effects unclear. Some of them may be ineffective, and the overuse of these medications may not only increase a patient’s burden and obscure the effectiveness of other medications but may be potentially harmful. The reasons why these drugs have been used for treating patients with AD in China are complicated. Instruction manuals mostly claim that they can treat memory disabilities, thus, confusing clinicians about whether to choose them or not. Additionally, such medications are listed as alternatives for treating AD in many textbooks, and only a few medical textbooks express objective opinions toward them. Thus, clinicians prescribe such medications for patients with AD who have memory disabilities, which does not compromise their medical principles. These medications are also easy to explain to patients and their family members. We think this is the main reason why many clinicians used such medications.

BPSD are common in patients with AD, and their incidence can be as high as 90 %[18]. The main symptoms are hallucinations, delusions, apathy, depression, agitation, and irritability [19]. The APA stated that “antipsychotic medications are recommended for the treatment of psychosis in patients with dementia”[10]. Apart from psychiatrists, other clinicians in different departments also are faced with patients with BPSD. Without a medical referral system in China, neurologists and GPs must prescribe these drugs themselves. That is the reason the results showed that all clinicians prescribed antipsychotic, antidepressant, and antianxiety agents. The results also showed there was a higher proportion of psychiatrists who combined the use of antipsychotics with other drugs than the proportions of neurologists and GPs that did so. The main reason may be that patients with BPSD might receive treatment at a psychiatric department, and psychiatrists have a better attitude toward antipsychotics than do neurologists and GPs.

Although typical and atypical antipsychotics are commonly used to treat dementia, the treatment effects of atypical antipsychotics are obviously better than the typical antipsychotics [20]. The FDA has warned against initially using Olanzapine and Risperidone with patients because of concerns about increased fatality rates while using them [21]. The present study showed that the three groups of clinicians mostly chose atypical antipsychotics, which were mainly Olanzapine and Risperidone. The reason may be that, although atypical antipsychotics have the same treatment effects as atypical antipsychotics, the atypical antipsychotics have fewer side-effects than the latter [22].

Due to restrictions on time, regions, and related resources, the present study has some limitations. First, the sample consisted of clinicians in Shanghai, so the sample is not representative of other regions of China. In addition, the questionnaire was completed based on the clinicians’ subjective memory recall, which may lead to certain recall biases. Future studies should be based on clinicians’ actual prescriptions in order to investigate these questions further.

## Conclusions

In conclusion, our study is the first study to report clinicians’ prescription preferences for treating patients with AD in China. Most clinicians had their own prescription preferences. For example, Huperzine A was widely used as a ChEI, however, the doses of ChEIs as well as Memantine were relatively small. Clinicians also tended to use cognitive enhancers and herbal medicines; yet there is not enough information about these agents from evidence-based research. Antipsychotics were mainly used by psychiatrists although they may have been beneficial for some patients who were treated by other clinicians. More regulations and training are needed for clinicians treating patients with AD.

## Appendix

**Table 5:** Hospital Distribution of the Clinicians in the Study

Hospital types	Psychiatrists (*n* = 39)	Neurologists (*n* = 66)	GP (*n* = 43)
Community hospitals, n (%)	7 (17.9 %)	0 (0)	21 (48.8)
Second-tier hospitals, n (%)	21(53.8 %)	18 (27.3 %)	8 (18.6 %)
Tertiary hospitals, n (%)	11 (28.2 %)	48 (72.7 %)	14 (32.6 %)
